# Learning from previous work and finding synergies in the domains of public and environmental health: EU-funded projects BRIDGE Health and HBM4EU

**DOI:** 10.1186/s13690-020-00460-9

**Published:** 2020-09-10

**Authors:** Madlen David, Gerda Schwedler, Lena Reiber, Hanna Tolonen, Anna-Maria Andersson, Marta Esteban López, Anke Joas, Miriam Schöpel, Alexandra Polcher, Marike Kolossa-Gehring

**Affiliations:** 1 German Environment Agency, Berlin, Germany; 2 Finnish Institute for Health and Welfare (THL), Helsinki, Finland; 3 Department of Growth and Reproduction, Copenhagen University Hospital (Rigshospitalet), Copenhagen, Denmark; 4 National Centre for Environmental Health, Instituto de Salud Carlos III, Majadahonda, Spain; 5 Ramboll Deutschland GmbH, Munich, Germany

**Keywords:** Human biomonitoring, HBM, Environmental health, Health information, HI, Health examination surveys, HES, HBM4EU, BRIDGE health, European projects, Public health, Indicators, Data repositories

## Abstract

**Background:**

During the last decade, the European Union initiated several projects in the domains of public and environmental health. Within this framework, BRIDGE Health (Bridging Information and Data Generation for Evidence-based Health policy and Research) and HBM4EU (European human biomonitoring initiative) have been implemented. Whereas, the focus of BRIDGE Health was towards a sustainable and integrated health information system (HIS), the aim of HBM4EU is to improve evidence of the internal exposure of European citizens to environmental chemicals by human biomonitoring (HBM) and the impact of internal exposure on health. As both, environmental and public health determinants are important for health promotion, disease prevention and policy, BRIDGE Health and HBM4EU have overlapping aims and outcomes. In order to improve health information regarding public health and environmental health issues, best use and exchange of respective networks and project results is necessary.

**Methods:**

Both projects have implemented health information (HI) and HBM tasks in order to provide adequate environmental and public health information of the European population. Synergies of the projects were identified in the working progress and because of overlapping networks and experts a focused analysis of both projects was envisaged. This paper elaborates on the aims and outcomes of both projects and the benefit of merging and channelling research results for the use of better health information and policy making that may be of relevance for any other project in these research fields.

**Results:**

The need for focused exchanges and collaborations between the projects were identified and benefits of exchanges were highlighted for the specific areas of indicator development, linkage of data repositories and the combination of HBM studies and health examination surveys (HES). Further recommendations for a European wide harmonisation among different tasks in the fields of public health and environmental health are being developed.

**Conclusions:**

Lessons learned from HBM4EU and BRIDGE Health show that continuous efforts must be undertaken, also by succeeding projects, to guarantee the exchange between public health and environmental health issues. Networks covering both are essential to provide better evidence of knowledge. The experiences from BRIDGE Health and HBM4EU give a valuable input for any future activity in these domains. Avoiding overlaps and streamlining further exchange of public health and environmental health contributes to best use of research results and allows to develop new strategies and tools for improvement of health information and thus enhances people’s health and well-being.

## Background

Public health is a major domain defined to prevent diseases, prolonging life and promoting health by targeted efforts from society [1]. Public health needs to include all determinants of health, also those studied in environmental health focusing on all the external factors (e.g. physical, chemical, biological) that may influence human health [2]. Direct exchange of both domains, public and environmental health, is essential to provide best knowledge on health information, but at present they are not consequently considered together. However, understanding the environmental determinants of health is a prerequisite to improve effective public health promotion and disease prevention policies.

In their everyday lives European citizens are exposed to numerous chemicals that are industrially produced or occur naturally in our environment. While not all substances pose a health risk, exposure to some can affect or even seriously damage human health [3]. Exposure of individuals may occur by inhalation, dermal and oral uptake from various sources as for example diet, consumer products, working and living environment and determines the body burden levels of every citizen [4]. Thus, the population is constantly exposed to a mixture of chemicals for which the health risks are still not fully understood.

For risk assessment in the context of environmental and public health, collective and individual exposure data for environmental substances are in increasing demand.

The internal body burden from exposure to environmental substances can be obtained using Human Biomonitoring (HBM). HBM is defined as the measurement of concentrations of substances or their metabolites in human biological matrices such as blood, urine or hair [5]. HBM integrates all routes of uptake and all relevant sources, as well as individual susceptibility. However, to be able to interpret HBM data in the light of health impact, health-based guidance values (HBM-GV) are required. HBM-GV for internal exposure such as the German HBM-values [6–8], European HBM-GV [9] and BE-values [10] allow to assess whether a concentration of a substance in the human body may pose a risk for certain health effects. Therefore, evaluating HBM data together with HBM-GV is an ideal tool for exposure and risk assessment for chemicals of concern. In the policy context, population based HBM data and evaluations are used to control if risk management actions have resulted in reduced health risk for European citizens or if new actions for further reduction are needed [8, 11].

In its 7th Environmental Action Programme (EAP), the European Union is aiming to assess and minimize environmental health risks for European populations from the use of hazardous chemicals by 2020 [12]. In order to reach this goal, several European projects were initiated over the last decades by the European Commission including environmental health by making use of HBM [13].

In 2015, BRIDGE Health^1^ (BRidging Information and Data Generation for Evidence-based health policy and research) was initiated by the Directorate General for Health and Food Safety (DG Sante) to integrate existing EU networks in the field of health information (HI), and to work towards a sustainable and integrated EU HI system. HI describes the health status and determinants of a population or a specific population group, and contains issues related to health care systems such as health system performance. Environmental health is also part of health information as a determinant of health.

Additionally, the EU provides funding for a broad range of projects and programmes covering different areas, also including the area ‘research and innovation’.

In 2017, the European Human Biomonitoring Initiative HBM4EU^2^ (Human Biomonitoring for Europe) was established by the European Commission, co-funded by Horizon 2020 – also an EU Research and Innovation programme This project is a joint effort with the aim to advance HBM in Europe in order to provide better evidence of the internal exposure of citizens from environmental substances to enhance knowledge in the domain environmental health [14].

HBM4EU builds directly on and expands the work of the 6th and 7th Framework Programme (FP6 and FP7) and the Life+ funding programme of the EU focusing on environment and climate action. Under these funding programmes also the HBM pilot projects ESBIO (Expert team to Support BIOmonitoring), COPHES (Consortium to Perform Human Biomonitoring on a European Scale) and DEMOCOPHES (Demonstration of a Study to Coordinate and Perform Human Biomonitoring on a European Scale) were co-financed from the EU which aimed to establish a long-term sustainable EU-wide HBM system as a science and policy tool [15, 16].

This paper evaluates and describes synergies between BRIDGE Health and HBM4EU. Based on observed synergies, recommendations to streamline action in both domains are suggested to enhance a European-wide harmonisation and better integration of environmental determinants of public health for improved health information.

## Materials and methods

Aims and outcomes regarding study and work focus of BRIDGE Health and HBM4EU were described and compared. Aims and outcomes of work packages, deliverables and publications of both projects were screened and synergies in the field of indicator development, interoperability of data and the combination of HES and HBM surveys were identified. Benefits of merging and exchanging scientific knowledge between the domains of public and environmental health were elaborated.

## Results and discussion

### Structure and scientific background of BRIDGE Health

BRIDGE Health ran from 2015 to 2017 with 31 participating institutions in 16 countries working towards a comprehensive, integrated and sustainable EU-HI structure, supporting an evidence-based health policy and research for the EU and its Member States (MS). Aim of the project was to bridge networks in domains of population and health system monitoring, indicator development, health examination surveys, environment and health, population injury and disease registries, clinical and administrative health data collection systems, and methods of health systems monitoring and evaluation.

The objectives of the BRIDGE Health project were:

- ensure sustainability of key HI activities that have been run under the previous health and research framework programmes of the EU and enhance synergy among these activities;
- enhance the transferability of HI for policy and improve the utility and use of data and indicators for stakeholders in policy making, public health surveillance and health care;
- reduce HI inequality within the EU and within MS;
- develop a blueprint for a sustainable and integrated EU HI system by developing common methods for standardization of HI gathering and exchange between population-based HI and health system information within and between MS.

**Fig. 1:**
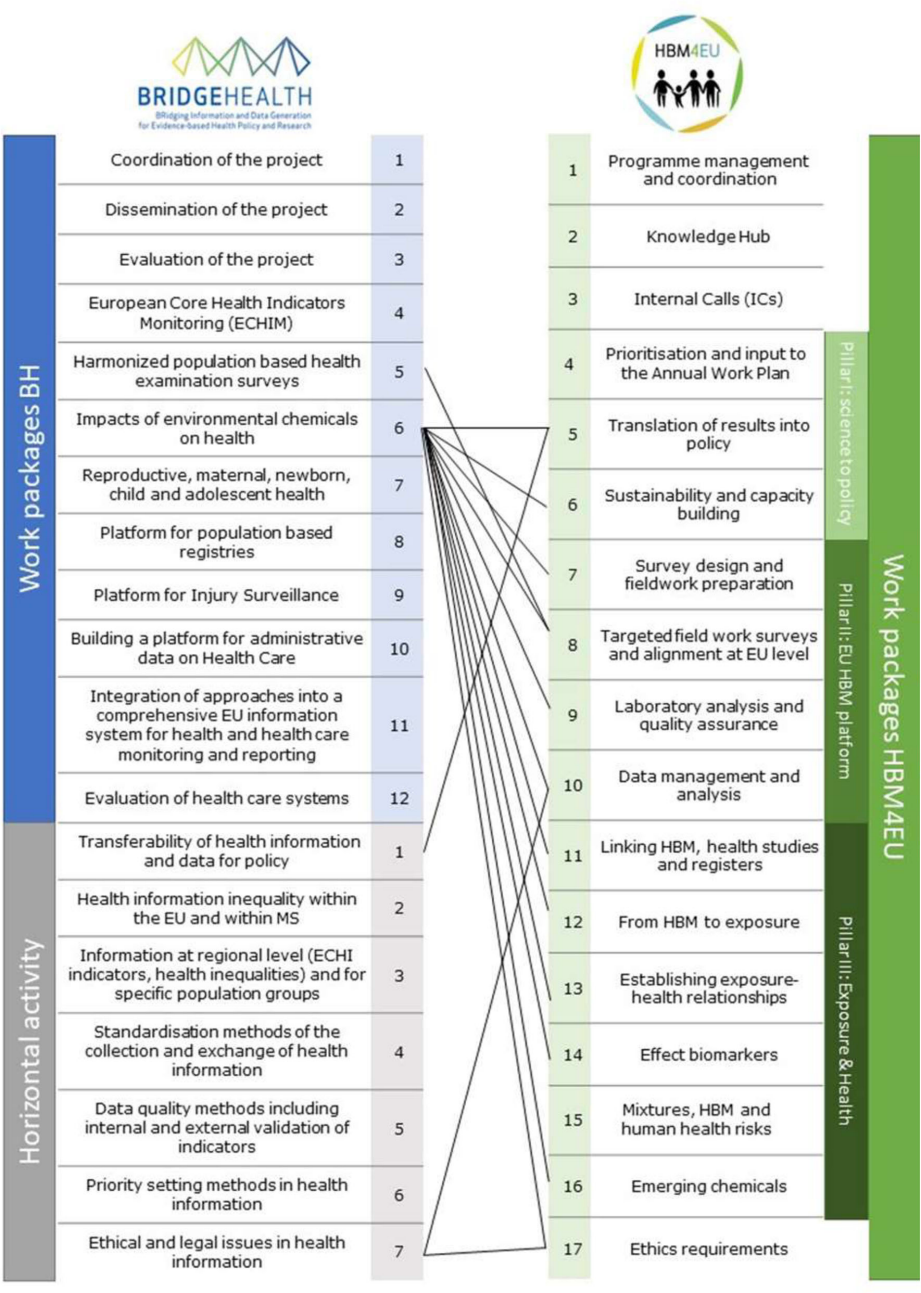
Structure and synergies of BRIDGE Health and HBM4EU

The work in BRIDGE Health was distributed over 12 Work Packages (WPs) and 7 Horizontal Activities (HAs) which addressed cross-sectional issues. An overview of the WPs and HAs of the project is given in Fig. 1.

There was a specific Work Package dedicated for issues of environmental chemicals and health, and the Horizontal Activity on priority setting methods in HI also included environmental health.

BRIDGE Health made use of 14 expert networks that contributed to the work and are summarized in Table 1. The networks comprise European and international experts from several domains of public health including environmental health to build on the best expertise for advanced research in bridging information and generating data for European HI. Consequently, HBM experts from COPHES [17, 18] were involved in the project, bringing the experiences how to harmonize HBM approaches in Europe to enhance better comparability of exposure burden from environmental substances of European citizens. Various other networks were involved in BRIDGE Health, for example experts running a database of European birth cohorts and experts for health indicators to make best use of research results and their communication to policy makers. The latter include health indicators like a cardiovascular surveillance set and the European Core Health Indicators (ECHI) that cover indicators assessing European health, the health care system and sanitation [19]. Another important partner within BRIDGE Health was the network EuroREACH which works on guarantees of comparability of health care data to enhance harmonisation within this sector. They built up an interactive platform for researchers, policy makers, and healthcare professionals to easily access health data and to enhance cross-country analysis of different European health systems. As there are all relevant networks from the fields of public and environmental health of Europe involved in BRIDGE Health, the project strengthened for the first time the exchange and harmonisation of expertise between HBM and HI tasks on a European level.

**Table 1:** Networks involved in BRIDGE Health

No	Network acronym	Network name	Link
1	COPHES	Consortium to Perform Human Biomonitoring on a European Scale	http://www.eu-hbm.info/cophes
2	CHICOS	Developing a Child Cohort Research Strategy for Europe	http://chicosproject.eu/
3	ENRIECO	Environmental Health Risks in European Birth Cohorts	https://www.enrieco.org/
4	EU-IBD	European Invasive Bacterial Disease Surveillance Network	https://ecdc.europa.eu/en/about-us/networks/disease-and-laboratory-networks/eu-ibd
5	EuroHOPE	European Health Care Outcomes, Performance and Efficiency	http://www.eurohope.info/
6	EUBIROD	EUropean Best Information through Regional Outcomes in Diabetes	http://www.eubirod.eu/
7	ECHO	European Collaborative for Healthcare Optimization	http://echo-health.eu/#
8	EUROCISS	European Cardiovascular Indicators Surveillance Set	http://www.cuore.iss.it/eurociss/en/project/project.asp
9	ECHIM	European Community Health Indicators and Monitoring	https://www.rki.de/EN/Content/Health_Monitoring/Projects/ECHIM/Echim_node.html
10	EHES	European Health Examination Survey	http://www.ehes.info/
11	EHLEIS	Advanced research on European health expectancies	http://www.eurohex.eu/index.php?option=welcome
12	EURO-PERISTAT	European Perinatal Information System	https://www.europeristat.com/
13	EuroREACH	access to health care data for cross-country comparisons of efficiency and quality	http://www.euroreach.net/home
14	Eurosafe	European Association for Injury Prevention and Safety Promotion	http://www.eurosafe.eu.com/home

### Structure and scientific background of HBM4EU

The European Human Biomonitoring Initiative HBM4EU (2017–2021) is a joint effort between 30 partner countries and the European Environment Agency (EEA). HBM4EU cooperates with an EU policy board consisting of five General Directorates (DG RTD, DG Sante, DG EMPL, DG ENV, DG AGRI, DG GROW, DG MARE and DG JRC) and three EU agencies, namely the European Environment Agency (EEA), European Chemicals Agency (ECHA), and European Food Safety Authority (EFSA). HBM4EU is organised around policy relevant questions for priority substances which were identified by the partner countries and the EU policy board. It aims to coordinate and further develop harmonised HBM and health studies at national and EU level, with a special focus on linking research to evidence-informed European policymaking, ensuring exploitation of results in the design of new chemicals policies and the evaluation of existing measures [14]. In addition, HBM4EU provides a robust interpretation of HBM data and the possible impact of environmental substances on human health. Communication of results and provision of data to policy makers, ensuring their exploitation in the design of new chemicals policies and the evaluation of existing measures, are major aims of the initiatives.

The key project objectives of HBM4EU are:

- harmonising procedures for HBM across the 30 participating countries to provide policy makers with comparable data on human exposure to chemicals and mixtures of chemicals at EU level;
- linking data on human internal concentrations of chemicals to aggregate external exposure and identifying exposure pathways and upstream sources;
- generating scientific evidence on the causal relation between human exposure to chemicals and health outcomes;
- providing relevant tools to detect emerging chemicals and to identify chemical mixtures of highest concern;
- adapting chemical risk assessment methodologies to use HBM data and account for the contribution of multiple external exposure pathways to the total chemical body burden;
- feeding information on exposure pathways into the design of targeted policy measures to reduce exposure.

These objectives are organised into 16 work packages clustered under three pillars shown in Fig. 1.

Data used and produced under HBM4EU will be made accessible via IPCHEM^3^ – the Information Platform for Chemical Monitoring. IPCHEM is the European Commission’s platform for searching, accessing and retrieving chemical occurrence data collected and managed in Europe.

Currently 117 institutes and around 450 experts are involved in HBM4EU (see Table 2), bringing together the whole expertise of HBM in Europe and expanding the European HBM network which was established within COPHES/DEMOCOPHES. Furthermore, HBM4EU builds up, for the first time in Europe, a centralised data management system within IPCHEM for HBM data and for further research to provide policy makers with the most recent knowledge on internal exposure burden of European citizens from environmental substances. Prioritised substances in the current research focus of HBM4EU are e.g. phthalates, per- and polyfluorinated substances, flame retardants, pesticides and other substances that are summarised under https://www.hbm4eu.eu/the-substances/. Working for the common aims, the project includes experts from universities, government authorities and research institutes from various fields of expertise such as medicine, toxicology, biology, statistics, occupational health, epidemiology, public health and food safety.

**Table 2:** Partners involved in HBM4EU sorted by country

No	Country	Institute	Link
1	Germany	German Environment Agency	https://www.umweltbundesamt.de/en
2		Institute for Prevention and Occupational Medicine of the German Social Accident Insurance	https://www.ipa-dguv.de/ipa/index-2.jsp
3		Helmholtz Center for Environmental Research	https://www.ufz.de/index.php?en=33573
4		Fraunhofer Institute	https://www.fraunhofer.de/en.html
5		German Federal Institute for Risk Assessment	https://www.bfr.bund.de/en/home.html
6		Friedrich-Alexander University Erlangen-Nürnberg	https://www.fau.eu/
7		Leipzig University	https://www.uni-leipzig.de/en/university/profile/portrait-of-leipzig-university/
8	Austria	Environment Agency Austria	https://www.umweltbundesamt.at/en/en_aboutus/about_us_eaa/
9		Medical University of Innsbruck	https://www.i-med.ac.at/mypoint/index.xml.en
10		Austrian Agency for Health and Food Safety	https://www.ages.at/en/ages/basics/
11		UMIT – Private University for Health Sciences, Medical Informatics and Technology	https://www.umit.at/page.cfm?vpath=universitaet/die-universitaet
12		Medical University of Vienna	https://www.meduniwien.ac.at/web/en/
13	Belgium	SCIENSANO	https://www.sciensano.be/en/about-sciensano
14		University of Liège	https://www.uliege.be/cms/c_8699436/en/uliege-portal
15		Flemish Institute for Technological Research	https://vito.be/en
16		Hasselt University	https://www.uhasselt.be/civic
17		The Flemish Region	https://www.belgium.be/en/about_belgium/government/regions/flemish_region
18		University of Antwerp	https://www.uantwerpen.be/en/
19		KU Leuven	https://nieuws.kuleuven.be/en/newsroom/media
20		Provincial Institute for Hygiene	https://www.provincieantwerpen.be/en/provincial-government/policy-areas.html
21	Switzerland	Swiss Tropical and Public Health Institute	https://www.swisstph.ch/en/
22		Federal Department of Home Affairs (FDHA)	https://www.edi.admin.ch/edi/en/home.html
23		Federal Department For Environment, Transport, Energy And Communications (DETEC)	https://www.uvek.admin.ch/uvek/en/home.html
24		Swiss School of Public Health	https://ssphplus.ch/
25		Alpine Foundation For Life Sciences	https://www.fasv.ch/
26	Cyprus	Ministry of Health of the Republic of Cyprus	https://www.moh.gov.cy/moh/moh.nsf/index_en/index_en
27		University of Cyprus	http://www.ucy.ac.cy/en/
28	Czech Republic	Masaryk University	https://www.muni.cz/en
29		Institute of Health Information and Statistics of the Czech Republic	http://www.uzis.cz/en
30		University of Chemistry and Technology Prague	https://www.vscht.cz/?jazyk=en
31		Institute of Experimental Medicine under the Czechoslovak Academy of Sciences	http://www.iem.cas.cz/en/
32		Charles University	https://cuni.cz/UKEN-1.html
33		St. Anne’S University Hospital BRNO	https://iweb3.fnusa.cz/?lang=en
34		Czech University of Life Sciences Prague	https://www.czu.cz/en/
35	Denmark	The Capital Region of Denmark	https://www.regionh.dk/english/Pages/default.aspx
36		University of Southern Denmark	https://www.sdu.dk/en/
37		Technical University of Denmark	https://www.dtu.dk/english/about
38		National Research Center for the Working Environment	http://nfa.dk/da/UK
39		University of Copenhagen	https://www.ku.dk/english/
40		Aarhus University	https://international.au.dk/
41		European Environment Agency	https://www.eea.europa.eu/
42	Greece	Aristotle University of Thessaloniki	https://www.auth.gr/en
43		University of Crete	http://www.en.uoc.gr/
44		The National and Kapodistrian University of Athens	https://en.uoa.gr/
45		Hellenic Health Foundation	http://www.hhf-greece.gr/index.html
46	Spain	Instituto Salud Carlos III	https://www.isciii.es/Paginas/Inicio.aspx
47		University of Las Palmas de Gran Canaria	http://www.english.ulpgc.es/
48		Barcelona Institute for Global Health	https://www.isglobal.org/en/
49		University of Granada	https://www.ugr.es/en/
50		Fundacio Institut d’Investigacio Sanitaria Pere Virgili (IISPV)	http://www.iispv.cat/en_index.html
51		Escuela Andaluza De Salud Publica SA	https://www.easp.es/
52		Foundation for the Promotion of Health and Biomedical Research of Valencia Region	http://fisabio.san.gva.es/en/fisabio
53	Finland	National Institute for Health and Welfare	https://thl.fi/en/web/thlfi-en
54		Finnish Institute of Occupational Health	https://www.ttl.fi/en/
55	France	Institut National De La Sante Et De La Rechercher Medicale	https://www.inserm.fr/en
56		French Alternative Energies and Atomic Energy Commission	http://www.cea.fr/english/Pages/cea/the-cea-a-key-player-in-technological-research.aspx
57		French National Centre for Scientific Research	http://www.cnrs.fr/en/cnrs
58		French National Institute for Agricultural Research	http://www.inra.fr/en
59		French Agency for Food, Environmental and Occupational Health & Safety	https://www.anses.fr/en
60		French National Institute for Industrial Environment and Risks	https://www.ineris.fr/en
61		French National Research and Safety Institute for the Prevention of Occupational Accidents and Diseases	http://en.inrs.fr/
62		Agence Nationale de Sante Publique	https://www.santepubliquefrance.fr/
63	Croatia	Croatian Institute of Public Health	https://www.hzjz.hr/en/
64		Institute for Medical Research and Occupational Health	https://www.imi.hr/en/
65	Ireland	Health Service Executive HSE	https://www.hse.ie/eng/
66	Israel	Ministry of Health	https://www.health.gov.il/English/Pages/HomePage.aspx
67	Iceland	University of Iceland	https://english.hi.is/university_of_iceland
68	Italy	Instituto Superiore di Sanita	https://www.iss.it/
69		Universita Degli Studi Di Napoli Federico II	http://www.unina.it/en_GB/home
70		University School for Advanced Studies IUSS Pavia	http://www.iusspavia.it/home
71		University of Udine	https://www.uniud.it/en/uniud-international/international-relations/the-university-of-udine
72		Azienda Sanitaria Locale Roma 1	https://www.aslroma1.it/
73		University of Modena	http://www.international.unimore.it/
74	Lithuania	National Public Health Surveillance Laboratory	http://www.nvspl.lt/
75		Lithuanian University of Health Sciences	http://www.lsmuni.lt/en/
76		Centre for Innovative Medicine	http://www.imcentras.lt/en/
77		Agency for Science, Innovation and Technology	https://mita.lrv.lt/en/
78	Latvia	State Education Development Agency	http://viaa.gov.lv/eng/
79		University of Latvia	https://www.lu.lv/en/
80		Rīga Stradiņš University	https://www.rsu.lv/en
81	Netherlands	National Institute for Public Health and the Environment	https://www.rivm.nl/en
82		Utrecht University	https://www.uu.nl/en
83		VU University Amsterdam	https://www.vu.nl/en/
84		Wageningen University and Research	https://www.wur.nl/en.htm
85		The Netherlands Organisation for Applied Scientific Research	https://www.tno.nl/en/
86		Radboud University	https://www.ru.nl/english/about-us/
87	Norway	Norwegian Institute of Public Health	https://www.fhi.no/en/
88	Poland	Nofer Institute of Occupational Medicine	http://www.imp.lodz.pl/home_en/
89	Portugal	Portuguese National Funding Agency for Science, Research and Technology	https://www.fct.pt/index.phtml.en
90		University of Lisboa - Faculty of Medicine	http://www.medicina.ulisboa.pt/en/
91		Instituto Nacional De Saude Dr. Ricardo Jorge	http://www.insa.pt/
92		Instituto Politecnico de Lisboa	https://www.ipl.pt/en
93		Ministerio da Saude - Republica Portuguesa	https://s-2.sns.gov.pt/
94	Sweden	Swedish Environmental Protection Agency	http://www.swedishepa.se/#
95		Karolinska Institutet	https://ki.se/en
96		Lund University	https://www.lunduniversity.lu.se/
97		Umeå University	https://www.umu.se/en/
98		Swedish National Food Agency	https://www.livsmedelsverket.se/en
99	Slovenia	National Institute of Public Health	https://www.nijz.si/en
100		Chemical Office of the Republic of Slovenia	http://www.uk.gov.si/en/
101		University Medical Centre Ljubljana	https://www.kclj.si/
102		Jožef Stefan Institute	https://www.ijs.si/ijsw/V001/JSI
103	Slovakia	Slovak Medical University in Bratislava	http://eng.szu.sk/
104		Constantine the Philosopher University in Nitra	https://www.ukf.sk/en/
105		Public Health Authority of the Slovak Republic	http://www.uvzsr.sk/en/
106		Slovak University of Technology in Bratislava	https://www.stuba.sk/english.html?page_id=132
107	United Kingdom	Public health England - Department of Health	www.dh.gov.uk
108		Brunel University London	https://www.brunel.ac.uk/
109		Institute of Occupational Medicine	https://www.iom-world.org/
110		United Kingdom Research and Innovation	https://www.ukri.org/
111		Health and Safety Executive	https://www.hse.gov.uk/
112	Hungary	Nemzeti Nepegeszsegugyi Kozpont	https://www.nnk.gov.hu/
113	Luxembourg	Laboratoire national de sante	https://lns.lu/en/
114		Luxembourg Institute of Science and Technology	https://www.list.lu/
115		Luxembourg Institute of Health	https://www.lih.lu/
116	North Macedonia	National Institute of Public Health	https://www.iph.mk/en/
117	Estonia	Estonian Health Board	https://www.terviseamet.ee/en/health-board

### Related work in BRIDGE health and HBM4EU

The work done in BRIDGE Health is closely related to the work undertaken by HBM4EU. Figure 1 shows the structure of both projects with their WPs and HAs and the identified synergies from the environmental health perspective, in order to highlight the exchanges and overlaps between the projects. Especially the work on the impacts of environmental chemicals on health done under BRIDGE Health is strongly linked to the work of HBM4EU. One of the aims in BRIDGE Health was to promote the use of environmental health surveillance as part of the European HI. Thus, the respective work package focused on contributing to a sustainable and integrated EU HI system regarding environmental determinants of health. It worked towards the identification of options to link HBM data with register information, the advantage of integrating HBM in health examination surveys (HES) and developed an indicator to assess impacts of environmental substances on health. The published work done within this working group stressed that there is a missing link between HI and environmental health surveillance [20]. Furthermore, the potential of environmental health surveillance and research data sources in the European population were evaluated to provide HBM-based indicators of internal human exposure and health impact of relevant chemicals [21].

Within HBM4EU, these tasks have been continued by integrating the expert team from the respective work package of BRIDGE Health. Two of the tasks within HBM4EU, where a clear knowledge transfer between the projects has been realised are the tasks to (a) evaluate the opportunities and obstacles of combining HBM and health studies and (b) to evaluate the availability of health studies, and administrative registers in different countries. Therefore, a report has been prepared at the beginning of HBM4EU to reflect on the coherences of BRIDGE Health and HBM4EU to develop further strategies of exchanging information [22]. Special focus of this Deliverable was on the use of HBM in public health policies and HI, on monitoring development in public and environmental health and on the valuable input of BRIDGE Health to enhance the work in HBM4EU in the respective fields of (a) combining HBM and HES, (b) indicator development and (c) linking data repositories.

BRIDGE Health focused on the optimisation of HES but did not yet include any alignment with HBM surveys. However, HES as well as HBM surveys share the important aspect of collecting health and lifestyle-related information of the study participants. This can be obtained via self-reported questionnaires as well as in specific physical measurements such as height, weight and blood pressure, and with the analysis of biological samples as done in a HES. The information about ongoing and planned national HES in the EU countries and the uses of HES data were collected and HES were assessed as highly valuable for health monitoring and for planning and evaluation of public health policies and prevention programmes [23].

BRIDGE Health also had specific tasks related to disease registries and injury surveillance which focused on the optimisation and harmonisation of registry set-up and data collection but did not yet investigate options to integrate additional parameters to allow the use in HBM interpretation and impact assessment of environmental stressors on health. However, these may be included in the future to ensure a synergistic data generation and data use for both environmental and public health policies.

BRIDGE Health had a special task on the transferability of HI and its data for policy. HI data and HI data platforms at national levels, within the EU, and around the globe have been made available. However, this data is mostly aggregated, and only poorly interlinked with each other. In addition, there are strong inequalities in data availability and quality, and a lack of appropriate means and formats for data transfer exists. Harmonisation of data, data transfer, and data protection rules are required. With the European General Data Protection Regulation (EU 2016/679), which came into force in 2018, an important step towards harmonisation of data protection of individual data and subsequent data transfer in the EU was taken.

### Examples for synergies

Three research fields with related content were identified in both projects (see Table 3). Better exchange and knowledge transfer of these fields may improve the picture of human health.

**Table 3:** Overview of synergies between the EU-projects BRIDGE Health and HBM4EU

Synergies	BRIDGE Health	HBM4EU
Indicator development	Revision of European Core Health Indicators (ECHI) for presenting relevant and comparable information on health at European level.	Development of HBM-based indicators for better knowledge transfer and policy advice of chemical exposure burden in the European population.
Interoperability of European databases	Establishment of EuroREACH working towards a comparative evaluation of health care systems in Europe.	HBM datasets will be fed into IPCHEM to support a more coordinated approach to collect, store and access monitoring data on chemicals and chemical mixtures, in humans and in the environment in Europe.
Combining HBM and HES	Update and further development of standardized protocols, related training materials, data management and reporting system for conducting HES at European level.	Elaboration of advantages for knowledge gain on human health in combining HBM and HES based on experiences from different countries. Implementation of feasibility studies to combine HBM studies and HES in Europe.

#### Indicator development regarding public and environmental health

HI is often expressed in form of indicators. The European Core Health Indicators (ECHI) Initiative started already in 1998 with several projects targeting the indicator development in the field of public health. Within ECHI framework, health indicators were developed with the aim to obtain a harmonised picture of the health conditions of European citizens [24–26]. Since then, the list of indicators has been continuously updated and revised - the last revision based on BRIDGE Health results was done in 2017 [27].

However, indicators that cover the field of environmental health and indicators based on HBM data are still rare worldwide. Only the ENHIS^4^ (Environment and Health Information System) indicators of the World Health Organisation (WHO) are actually using HBM methods to collect data. In the ENHIS list there are two indicators based on HBM, namely ‘blood lead levels in children’ and ‘POP (persistent organic pollutants) levels in human milk’. Other available indicators in the field of environmental health are proportion of daily smokers, consumption of fruit and vegetables, work-related health risks and particulate matter (PM) exposure. In BRIDGE Health the possibilities to link information from HBM with register information (environmental databases, perinatal health registers, chronic disease registries) as well as with health indicators were elaborated. Furthermore, similarities and differences in data collection and data management were assessed in order to derive recommendations for future work towards the integration of HBM in HI [20]. In the context of integrating HBM in the HI system, procedural steps and an outlook for developing HBM-based indicators to evaluate internal body burden from environmental substances on health were given. Consequently, and based on this, the work on policy relevant HBM indicators for chemical exposure was continued within HBM4EU. In general, indicators should be seen as a tool to facilitate communication between science, policy and the public. Hence, indicators should contain simple messages related to the internal exposure of environmental substances in citizens and should be understandable for everybody [28]. Two types of indicators were developed and tested in the framework of HBM4EU: (a) HBM indicators of internal exposure representing exposure burden of a substance or group of substance in the population and (b) HBM indicators for health risks showing to which degree certain health-based guidance values (HBM-GV) are exceeded. Up to now, two hazardous chemicals/group of chemicals were used as first examples for both types of indicators: bisphenol A (BPA) and per- and polyfluoroalkyl substances (PFAS). Further work on indicators will be continued within HBM4EU on phthalates, cadmium, bisphenol A, PFAS (PFOA and PFOS) and remaining sets of priority chemicals.

#### Interoperability of European databases

HI as well as HBM data were collected and stored in different databases mainly on national level, but both projects focused on bringing this information on a European level. Therefore, new centralised data repositories for each of the fields, HI and HBM, have been established separately but have not been linked to each other. However, linking individual data repositories is an important approach to ensure that all available data can be used for the research to maximise their scientific potentials. For the improvement of the use of data repositories in public health the EuroREACH project was established within BRIDGE Health to provide a toolbox of guidance to researchers, policy makers and other stakeholders interested in cross-country research by:

- Identifying information sources of aggregated, patient-level, and disease-based data
- Offering guidance on key data challenges such as data access, linkage and comparability
- Highlighting gaps in existing data to encourage data collection in under-represented areas.

Finally, with EuroREACH a first step towards comparative evaluation of health care systems in Europe was made and a centralised database was built.

In contrast, HBM4EU was initiated to provide country comparisons of exposure burden from environmental substances in Europe. Therefore, making data interoperable and increasing data re-use are two important tasks of the project. Already existing data on a national level as well as on European level have been merged in one database in order to make data more easily available for different users. A data management plan was established in HBM4EU guiding the researchers on how existing and newly generated data need to be processed, managed, and quality controlled at the national level, and subsequently harmonised, transferred and analysed at EU-level [29]. A common definition and code list of pre-defined values for harmonising the descriptions of HBM metadata and data is a further challenge of HBM4EU. The transfer of available HBM4EU data into the European Commission’s IPCHEM, which is managed by the Joint Research Centre (JRC) has already been achieved for metadata and aggregated data. New exposure data will be collected continuously, harmonisation and comparability of data will be guaranteed. Datasets will be made publicly available on the IPCHEM website for further research topics, for the public or for policy makers in accordance with the European General Data Protection Regulation (EU 2016/679).

#### Combining human biomonitoring (HBM) and health examination surveys (HES)

In BRIDGE Health, HES activities were investigated in detail [23] and also the study design of HBM surveys was elaborated [20]. HBM and HES share a lot of common aspects with the potential to be combined and harmonised to build on comprehensive and consolidated population health surveys. Both kind of surveys are examination surveys based on interviews and measurements. Whereas HBM surveys have their study focus on exposure burden from environmental chemicals in humans measured in biological samples such as urine or blood, HES are mainly focussed on assessing human health with different measurements, like BMI, blood pressure, breathing volume (spirometry) and clinical biomarkers in blood or urine reflecting health. However, the structure of both survey types is very similar and they share common tools such as recruitment, questionnaires, collection of biological samples, data collection, quality measures and collect both personal information such as age, sex and the socioeconomic status. Both survey types follow good epidemiological practice, including ethics and data protection issues. The identified similarities and the overlaps in data and sample collection suggest a combination of HES and HBM studies if it is feasible from an organisational point of view. This combination reduces the efforts and costs required for the logistic in the field work and allows to achieve a more complete information of the target population, giving the possibility of exploring the link between internal body burden and health effects. Main obstacles were identified in the lack of flexibility of data access between databases and limitations of the size of questionnaires to minimize time effort for participants and to maintain their willingness to participate. Work done in HBM4EU for preparing guidelines to combine HBM and health studies builds on the expertise and experience from BRIDGE Health. Some of the EU or EU associated countries have already combined HBM with HES surveys, like Germany, France and Israel. In France the Esteban Survey ran from 2014 to 2016 and in Israel two combined surveys run in 2011 and in 2015 to 2016, respectively [30, 31]. The longest tradition for conducting combined HBM and HES however exists in Germany: since 1985 five German Environmental Surveys (GerES) were conducted in combination with respective HES surveys [32, 33]. Currently, GerES VI is in preparation in cooperation with the respective HES. The field work is planned to start in 2021. The experiences of France, Israel and Germany were used within HBM4EU to further develop the combination of HBM and HES studies in order to make the implementation of a combined HBM and HES survey feasible for more countries in the future. Moreover, a detailed work programme to investigate the opportunities and obstacles of combining on-going and planned health studies (HES, cohort studies, dietary surveys, occupational studies, etc.) and HBM studies was prepared within the report [34]. Experiences from the countries mentioned above have been collected and evaluated. HBM and HES protocols were compared with the purpose of identifying commonalities and differences of HBM studies and HES phases and contents. Based on this knowledge a guideline was elaborated within HBM4EU which allows countries to combine HBM and HES surveys in an integrative way. Countries, where HES and HBM surveys are already jointly conducted, already contribute to further capacity building for population health surveys including environmental health. Benefits and lessons learned from these combined surveys may help to advance research for public and environmental health.

### Future steps towards a European HI system including environmental health

During the last years, HES and HBM in Europe underwent a great development as additional efforts are needed in order to bring national projects on European level. Important challenges have to be solved since there are political, geographical and other differences that impede the implementation of HES and HBM surveys in Europe. However, collaboration among experienced scientists from Centers for Disease Control and Prevention (CDC) and others (e.g. Health Canada) has been a valuable support to improve and boost HBM at European level.

The BRIDGE Health focus was mainly on HI data sources and tools, such as HES, health registries, and indicator development but also covered environmental health including HBM experts. The work on establishing a sustainable health information system for EU has been continued in the follow-up of BRIDGE Health under the framework of the Joint Action on Health Information^5^ (InfAct), which was launched in March 2018 and aims to establish a sustainable health information framework for the EU [35]. Both InfAct and HBM4EU have a strong focus on policy advice through evidence-informed decision making. A continuous exchange of health and environmental data between HBM4EU and InfAct will be a benefit in research for both projects. Both projects build on a significant level of available expertise, use of existing national infrastructures and integration of networks. HBM4EU and InfAct will run until 2021 and it is very likely that follow-up projects will continue their work in the future. Lessons learned from both projects should be considered in order to combine public and environmental health with particular focus on indicator development, the interoperability of repository platforms and the combination of HBM studies and HES. Combining indicator lists in the fields of public and environmental health may advance the knowledge of human health by implementing chemical exposure burden and may consequently improve health information and targeted policy making. As a basis for the development of indicators the exchange or aggregation of repository platforms of both fields may help to give a more valuable and consolidated output of research data. For gathering data for both needs, it can be recommended to continuously combine HES and HBM surveys to build up profound and stable research studies including the needs of public and environmental health. In conclusion, exchanging research activities and datasets from HBM and HI in the future may complete the picture of human health and may provide an important support for countries worldwide with less experience and resources that have to face up challenging situations regarding human health and environmental pollution.

## Conclusions

BRIDGE Health, its follow-up InfAct and HBM4EU are major projects in the fields of public and environmental health in Europe. Between these projects, various relationships and synergies exist. Indicator development, interoperability of repository platforms (like IPCHEM) as well as capacity building for population health (HES) and environmental surveys (HBM surveys) are three important topics, that show how results and conclusions of one project can be incorporated into following projects. Experiences from BRIDGE Health and HBM4EU give a valuable input for any further EU-project in the domains of public or environmental health. Lessons learned from HBM4EU and BRIDGE Health show that continuous efforts must be undertaken to guarantee the exchange between both domains, for example in the frame of InfAct, any subsequent HI research network or a HBM4EU follow-up project. Learning from each other and working on harmonisation is a continuous task and will bring added value for providing well-founded health information.

Further exchange and progress on HI as well as on HBM improve both, public health and environmental health and thus contribute to foster people’s health and well-being in Europe and beyond.

## Data Availability

It is envisaged that Human biomonitoring datasets generated and/or analysed during HBM4EU will be made accessible in the IPCHEM platform, see https://ipchem.jrc.ec.europa.eu/RDSIdiscovery/ipchem/index.html.

## Abbreviations

BE: Biomonitoring Equivalents
BPA: bisphenol A
BRIDGE Health: Bridging Information and Data Generation for Evidence-based Health Policy and Research
CDC: Centers for Disease Control and Prevention
COPHES: Consortium to Perform Human Biomonitoring on a European Scale
DEMOCOPHES: Demonstration of a Study to Coordinate and Perform Human Biomonitoring on a European Scale
DG AGRI: The Commission’s Directorate-General for Agriculture and Rural Development
DG EMPL: The Commission’s Directorate-General for Employment, Social Affairs and Inclusion
DG ENV: The Commission’s Directorate-General for Environment
DG GROW: The Commission’s Directorate-General for Internal Market, Industry, Entrepreneurship and SMEs.
DG JRC: The European Commission’s in house science service - Joint Research Centre
DG MARE: The Commission’s Directorate-General for Maritime Affairs and Fisheries
DG RTD: The Commission’s Directorate-General for Research and Innovation
DG Sante: The Commission’s Directorate-General for Health and Food Safety
ECHA: European Chemicals Agency
ECHI: European Core Health Indicators
EEA: European Environment Agency
EFSA: European Food Safety Authority
ENHIS: Environment and Health Information System
HA: Horizontal Activity
HBM: human biomonitoring
HBM4EU: Human Biomonitoring Initiative
HBM-GV: European health-based guidance values
HES: health examination surveys
HI: health information
InfAct: Information for Action!
IPCHEM: Information Platform for Chemical Monitoring
PFAS: per- and polyfluoroalkyl substances
PFOA: perfluorooctanoic acid
PFOS: perfluorooctane sulfonic acid
WP: Work Package

## Footnotes

1 http://www.bridge-health.eu/

2 https://www.hbm4eu.eu/

3 https://ipchem.jrc.ec.europa.eu/RDSIdiscovery/ipchem/index.html

4 https://gateway.euro.who.int/en/datasets/enhis/

5 https://www.inf-act.eu/
